# Droplet Impact on the Super-Hydrophobic Surface with Micro-Pillar Arrays Fabricated by Hybrid Laser Ablation and Silanization Process

**DOI:** 10.3390/ma12050765

**Published:** 2019-03-06

**Authors:** Zhenyan Xia, Yuhe Xiao, Zhen Yang, Linan Li, Shibin Wang, Xianping Liu, Yanling Tian

**Affiliations:** 1 School of Mechanical Engineering, Tianjin University, Tianjin 300350, China; xia_zhy@tju.edu.cn (Z.X.); xiaoyuhe@tju.edu.cn (Y.X.); lali@tju.edu.cn (L.L.); shbwang@tju.edu.cn (S.W.); 2 Key Laboratory of Mechanism Theory and Equipment Design, Ministry of Education, Tianjin University, Tianjin 300350, China; 3 School of Engineering, University of Warwick, Coventry CV4 7AL, UK; X.Liu@warwick.ac.uk

**Keywords:** nanosecond laser, super-hydrophobic, droplet impacting, maximum spreading factor, viscous dissipation

## Abstract

A super-hydrophobic aluminum alloy surface with decorated pillar arrays was obtained by hybrid laser ablation and further silanization process. The as-prepared surface showed a high apparent contact angle of 158.2 ± 2.0° and low sliding angle of 3 ± 1°. Surface morphologies and surface chemistry were explored to obtain insights into the generation process of super-hydrophobicity. The main objective of this current work is to investigate the maximum spreading factor of water droplets impacting on the pillar-patterned super-hydrophobic surface based on the energy conservation concept. Although many previous studies have investigated the droplet impacting behavior on flat solid surfaces, the empirical models were proposed based on a few parameters including the Reynolds number (*Re*), Weber number (*We*), as well as the Ohnesorge number (*Oh*). This resulted in limitations for the super-hydrophobic surfaces due to the ignorance of the geometrical parameters of the pillars and viscous energy dissipation for liquid flow within the pillar arrays. In this paper, the maximum spreading factor was deduced from the perspective of energy balance, and the predicted results were in good agreement with our experimental results with a mean error of 4.99% and standard deviation of 0.10.

## 1. Introduction

Surface wettability is one of the significant properties for a particular solid substrate [1]. Inspired by natural lotus leaves [2], rose petals [3], and butterfly wings [4], super-hydrophobic surfaces (i.e., apparent contact angle above 150° and sliding angle below 10°) have been successfully mimicked through the synergetic effects of micro/nanostructure fabrication and surface chemical modification [5,6,7,8,9,10]. Due to their enormous potential applications including anti-icing [11], drag reduction [12], self-cleaning [13], anti-bacteria [14], and corrosion resistance [15], super-hydrophobic surface mimicry has been extensively developed by state-of-the-art techniques, such as thermal imprinting [16,17], chemical vapor deposition [18], coating [19], electrochemical deposition [20,21], and laser texturing [22,23,24,25,26,27]. Particularly, laser texturing can be seen as one of the facile approaches, and therefore can be extensively utilized to fabricate super-hydrophobic substrates owing to its precise control of surface fabrication with three dimensional (3D) hierarchical structures [28].

Droplet impact on solid surfaces is an important element of various phenomena encountered in practical applications, including ink-jet printing, crop spraying, internal combustion engines for hydrophilic/super-hydrophilic surfaces, and self-cleaning and anti-icing for hydrophobic/super-hydrophobic surfaces [29]. Given that the liquid droplet is in a dynamic state when impacting on a rigid surface, the contact process between the droplet and the rigid surface should be explored in detail because it plays a significant role in energy conversion between the thermal energy and kinetic energy [30]. In particular, a droplet impacting on a super-hydrophobic surface will firstly spread to a maximum diameter and then recoil to a specific extent that it can entirely rebound and leave the solid substrate. During the impacting, it has been proved that the contact time, surface interactions, internal energy dissipation, inertia, and capillarity of the droplet are of great importance to assess the performance of a super-hydrophobic surface for its anti-icing and self-cleaning functions [31,32]. Besides, the maximum spreading factor (i.e., the ratio of the maximum spreading diameter (*D*_max_) to the initial diameter (*D*_0_) of the droplet, *β*_max_ = *D*_max_/*D*_0_) is always an important parameter of interest because it can normalize the maximum deformation of a droplet [33].

Previous studies have validated that the relevant parameters determining the maximum spreading factor are the Reynolds number (*Re* = *ρD*_0_*V*_0_/*μ*), Weber number (*We* = *ρD*_0_*V*_0_*^2^*/*σ*) [34], Ohnesorge number (*Oh* = *We*^1/2^/*Re*) [35], as well as the capillary number (*Ca* = *We*/*Re*) [36], where *V*_0_ and *D*_0_ are the initial impact velocity and diameter of the liquid droplet. The liquid density, surface tension, and viscosity are denoted by *ρ*, *σ*, and *μ*, respectively. Based on these parameters, previous empirical prediction models of the maximum spreading factor were obtained via simply simulating the experimental data. For instance, Scheller et al. [37] investigated the maximum spreading diameter of the impacting droplet with a wide range of velocities and liquid viscosities, and then an empirical model was proposed based on the *Re* and *Oh* number. Seo et al. [38] explored the impacting behavior of gasoline and isooctane, and the maximum spreading factor was obtained by modifying the coefficients of Scheller’s model. Beyond that, according to the concept of energy conservation between pre-impacting state and the maximum spreading state, Chandra et al. [39] and Passandideh-Fard et al. [40] took the contact angle into consideration when proposing their models to simulate the maximum spreading factor. Mao et al. [41] systematically investigated the influences of viscosity, impact velocity, and surface roughness on the impacting process, and the formula of maximum spreading factor was acquired as a function of *Re*, *We*, and contact angle. However, it is noted that the abovementioned prediction models were obtained for the flat solid surfaces, which may not be applied to the super-hydrophobic surfaces, especially a super-hydrophobic surface with pillar arrays.

Recently, the insights into droplet impact on the super-hydrophobic surfaces have attracted extensive attention due to the distinguished bouncing phenomenon compared with the flat hydrophilic surfaces, which can provide valuable solutions to design the self-cleaning and anti-icing devices. For example, Malla et al. [42] mainly investigated the surface morphology and Weber number on the droplet dynamics and impact outcome (i.e., no bouncing, complete bouncing, and bouncing with droplet breakup). Lee et al. [43] demonstrated that two distinct Cassie-to-Wenzel wetting transitions occurred during the droplet impacting the super-hydrophobic surface: one taking place right after contact, and the other during the droplet retraction before rebound. Besides, Guo et al. [44] focused on the investigation of contact time and drop shape of the impacting droplets upon the anisotropic super-hydrophobic surfaces. However, the author noticed that the maximum spreading factor of water droplets on the super-hydrophobic surfaces was seldom reported. In fact, the maximum spreading factor on the super-hydrophobic surfaces was definitely different from the flat surfaces due to the variation of wetting states which results in the difference of viscous energy dissipation during the impacting process. It is therefore urgent to explore the maximum spreading factor and propose a prediction model for super-hydrophobic surfaces, which can promote their practical application in the fields of anti-icing and self-cleaning.

In this paper, a pillar-patterned surface was fabricated by the nanosecond laser ablation, and the (heptadecafluoro-1,1,2,2-tetradecyl) triethoxysilane (FAS) was further used to reduce the surface free energy and render the laser-induced surface to show super-hydrophobic character. The generation mechanism of the super-hydrophobicity was explored though analyzing the surface morphologies and surface chemical compositions. Then, the liquid droplet impacting on this super-hydrophobic surface was recorded utilizing a high-speed video camera, and its maximum spreading diameter was acquired. Moreover, the theoretical prediction model of maximum spreading factor was proposed by considering the geometrical parameters and two sources of viscous energy dissipation on the super-hydrophobic surface: one comes from the surface interaction between the liquid film and the top areas of the pillars and the other comes from the liquid flowing among the pillar arrays. The computational results demonstrated that the present model was more accurate within a 5% mean error to predict the maximum spreading diameter on the as-prepared super-hydrophobic surface. The developed model and obtained results can not only promote the theoretical analyses of droplets impacting on the super-hydrophobic surfaces with pillar patterns, but also enhance the practical applications of solid surfaces in the field of anti-icing, self-cleaning, and spray coating.

## 2. Materials and Methods

### 2.1. Materials

A 6061 aluminum alloy with a dimension of 50 mm × 30 mm × 5 mm was explored in this study. In order to ensure surface uniformity, #2000 silicon carbide abrasive papers were used to mechanically polish the samples. Subsequently, the finished surfaces were chemically washed in ultrasonic bath with the cleaning solutions of acetone, ethanol, and deionized water in sequence each for 5 min. Then the rinsed samples were dried by compressed nitrogen gas flow.

### 2.2. Fabrication of Micro-Pillar Arrays

A nanosecond fiber laser (wavelength 1064 nm, output power 10 W, repetition rate 20 kHz, pulse duration 50 ns, and spot size 50 μm, IPG photonics, Brubach, Germany) was applied to fabricate the ordered array of micro-pillar structures on the prepared substrates. This laser machine was equipped with an X/Y scanner and a focusing lens. Installed on the working platform, the substrates were ablated by the moving laser beam, first along the *x* (0°) then along the *y* (90°) directions. The distance between adjacent laser scanning paths was set at 150 μm and the laser scanning speed was set at 500 mm/s. As a result, the micro-pillar arrays were obtained on the prepared samples.

### 2.3. Chemical Modification with FAS

The laser ablated surfaces with micro-pillar arrays were immersed into a mixed solution containing distilled water, ethanol and (heptadecafluoro-1,1,2,2-tetradecyl) triethoxysilane (FAS) for three hours at room temperature. Deionized water was applied to flush the prepared samples, and then they were dried at 60 °C in a commercial oven for one hour. Finally, the processed surfaces exhibited a super-hydrophobic character.

### 2.4. Measurement and Characterization

**Figure 1:**
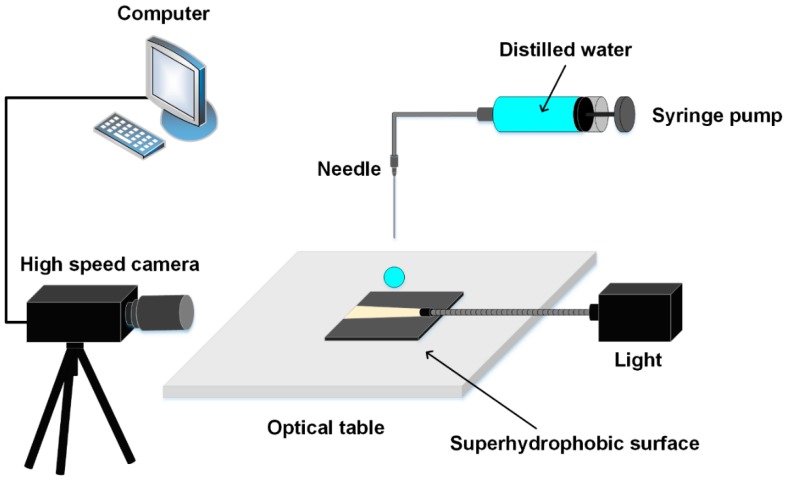
Schematic for the droplet impacting experiment.

Helions G4 CX environmental SEM (FEI, Hillsboro, OR, USA) and CountourGT 3D optical microscope (Bruker, Tucson, AZ, USA) were utilized to observe the surface topographies. An Escalab 250Xi XPS was used to evaluate the surface chemical compositions (Thermo Fisher Scientific, Waltham, MA, USA). The apparent contact angle (APCA) and sliding angle (SA) measurements were conducted by a VCA optima instrument (AST, Billerica, MA, USA) using the water droplet with a volume of 6 μL at the temperature of 25 °C. A HX-3E high-speed video camera (NAC, Hokkaido, Japan) was used to record the dynamics of the droplets at the frequency of 6000 frames per second. A high-intensity visible light source (S5000 Hecho, Nanjing, China) was used to generate background light. The schematic of droplet impacting experiments is shown in Figure 1.

## 3. Results and Discussion

### 3.1. Surface Microstructures

**Figure 2:**
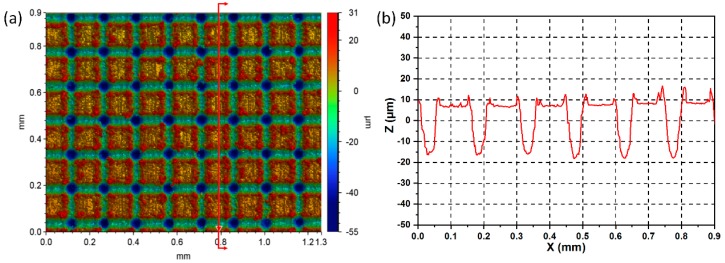
(**a**) 3D and (**b**) cross-sectional profilers of the super-hydrophobic surface.

**Figure 3:**
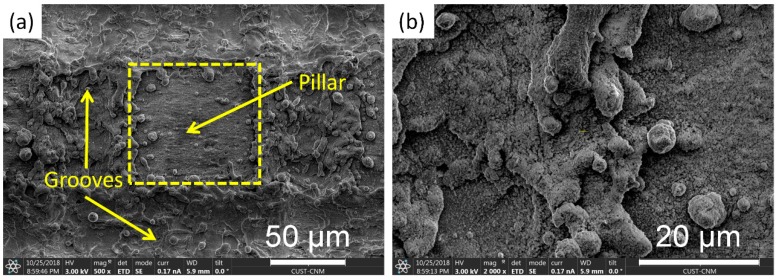
The morphology of the super-hydrophobic surface after laser ablation treatment. (**a**) Magnification of 500×; (**b**) Magnification of 2000×.

Surface morphologies of the super-hydrophobic surfaces were studied using the 3D optical microscope and SEM, as shown in Figure 2 and Figure 3. It can be seen from Figure 2a that this functional surface is remarkable for micro-pillar architecture and micro-grooved surface pattern. Figure 2b shows that the average depth and width of the micro-grooves were 23.9 μm and 65.1 μm, respectively. As shown in Figure 3a, this SEM image shows that the aluminum alloy surface was intensively heated and then melted along the focused laser beam, resulting in the formation of microgrooves. Owing to the rapid cooling effect, the splashed materials were quickly solidified and covered on the brims of the laser-induced grooves, as shown in the enlarged SEM image of Figure 3b. After laser scanning with two perpendicular directions, the ordered surface texture with micro-pillar arrays was fabricated. Therefore, the micro-pillars patterned surface with many micro/nano-scaled particles (i.e., the hierarchical rough surface structures) was received after laser ablation treatment. This unique surface texture can considerably enhance specific surface area, which can facilitate the attachment of functional groups originating from the FAS molecules.

### 3.2. Surface Chemistry

**Figure 4:**
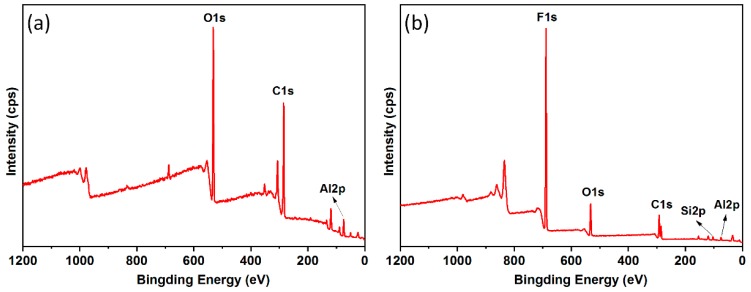
XPS survey spectra of (**a**) polished aluminum alloy and (**b**) as-prepared super-hydrophobic surface.

Due to large content of functional groups with low-free-energy (i.e., –CF_2_ and –CF_3_), FAS was widely used to modify surface chemical compositions on the roughed surface structures. X-ray photoelectron spectroscopy (XPS) spectra of the polished substrate and as-fabricated super-hydrophobic surface are displayed in Figure 4. It is obvious that two additional F 1s and Si 2p peaks were observed on the laser-induced super-hydrophobic surface after chemical modification of FAS. The deconvolutions of C 1s and O 1s were performed by the CasaXPS software (Version 2.3.19, Casa Software Ltd.) to provide further insight into the formation mechanism of the super-hydrophobicity.

**Figure 5:**
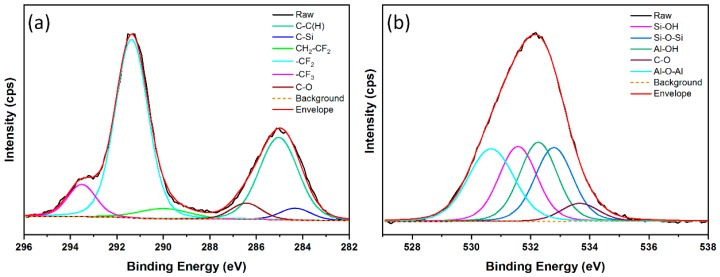
The high-resolution spectra of (**a**) C 1s and (**b**) O 1s.

The high-resolution spectra of C 1s is displayed in Figure 5a. The C 1s peak for the super-hydrophobic surface was composed of six main contributions: C1 locating at 284.4 eV that corresponded to the C–Si bond [45]; C2 at 285.0 eV that was contributed to by the C–C and C–H bonds; and C3 locating at 286.4 eV that was due to the C–O bind. Particularly, C4, C5, and C6 denote the functional bonds of –CH_2_–CF_2_–, –CF_2_–CF_2_–, and –CF_3_, whose binding energy were centered at 290.1 eV, 291.4 eV, and 293.4 eV, respectively. The detected functional groups confirmed that the FAS chains were successfully assembled on the laser-produced surface. The formation mechanism can be described as follow. In the first step, FAS molecules will be hydrolyzed when reacting with water, producing the Si–OH functional group. In the next step, the dehydration effect will occur between the Si–OH and Al–OH bonds. The large amount of Al–OH originated from the hydroxylation effect because the laser-induced alumina can react with the interfacial water vapor molecules. Therefore, due to the dehydration effect, the long chains of FAS molecules were assembled on the produced rough surface. As a result, the surface free energy can be remarkably lowered owing to the existence of –CF_2_ and –CF_3_ functional groups.

The deconvolution of O 1s was further conducted to confirm the formation mechanism of the super-hydrophobicity. As shown in Figure 5b, the O 1s peak was decomposed to five components, of which locating at 530.9 eV and 532.3 eV were related to the lattice oxygen peak (Al–O–Al) and oxygen peak associated with hydroxyls (Al–OH) [46]. In addition, the other three peaks at 531.5 eV, 532.8 eV, and 533.8 eV corresponded to the bonds of Si–OH, Si–O–Si, and C–O, respectively. These three functional groups clearly indicated that the FAS chains were assembled on the laser-induced surface. Specifically, the existing of the Si–O–Si bond revealed that a special cross-linked structure was created between two FAS molecules.

By analyzing the surface chemistry, the generation mechanism of the super-hydrophobicity could be proposed. The laser-induced rough structures with the ordered micro-pillar arrays enhanced the specific surface area. After modification of the FAS, the functional groups with low-free-energy were attached on the laser-induced surface texture. Therefore, the super-hydrophobicity was related with the synergetic effects of rough surface structure and the low-free-energy fluorinated functional groups.

### 3.3. Wettability

**Figure 6:**
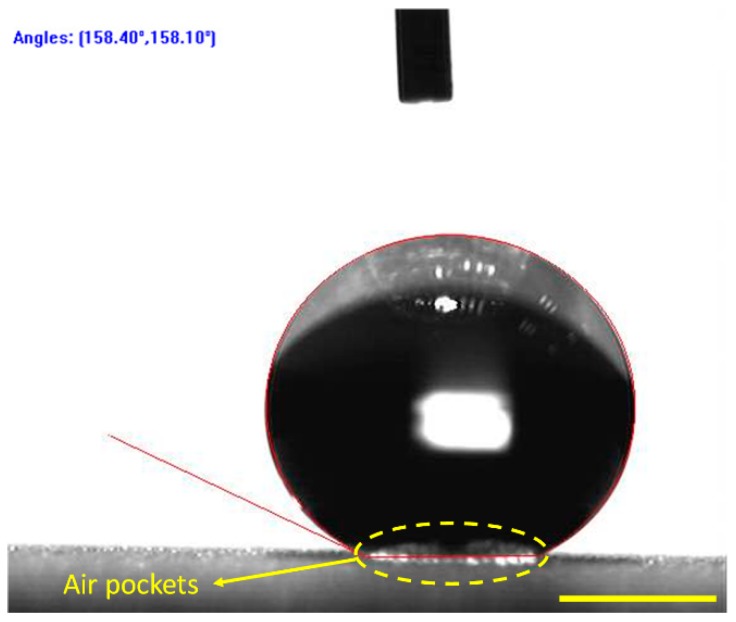
Optical picture of a 6 μL water droplet on the fabricated substrate. The scale bar is 1 mm.

Figure 6 shows the optical picture of a 6 μL water droplet placed on the as-prepared super-hydrophobic aluminum alloy surface. It is clearly observed that the shape of the water droplet almost exhibits the entire sphere, indicating that the as-prepared surface showed minimal pining effect. It can be verified by its high APCA ~158.2 ± 2.0° and low SA ~3 ± 1°. Meanwhile the advancing and receding contact angles of the super-hydrophobic surface were averaged at 159.8° and 156.1°, respectively. Due to the existence of surface pillars and the low-free-energy film, many air pockets were trapped underneath the liquid droplet. It can be proved from Figure 6 that many bright air pockets were observed underneath the water droplet, resulting in the droplet suspension on the as-prepared surface. Because the air is absolutely hydrophobic, the amount of trapped air can effectively promote surface hydrophobicity [47].

### 3.4. Droplet Impacting Behavior

In this study, the distilled water with the diameter *D*_0_ ≈ 2.57 mm was used to conduct the impacting experiments. It is reported that the durability of FAS layers on the super-hydrophobic aluminum alloy surfaces will be gradually deteriorated upon long-term immersion in water [48]. In order to avoid any degradation of hydrophobic property, the droplet impacting experiments were carried out on different locations of the as-prepared super-hydrophobic surfaces. The velocities varied from 0.37 to 1 m/s by changing the droplet release height compared with the reference surface of the super-hydrophobic samples. The corresponding Weber number and Reynolds number were 5 ≤ *We* ≤ 35 and 938 ≤ *Re* ≤ 2534, respectively. All the impacting experiments were carried out in the cleanroom at normal temperature.

**Figure 7:**
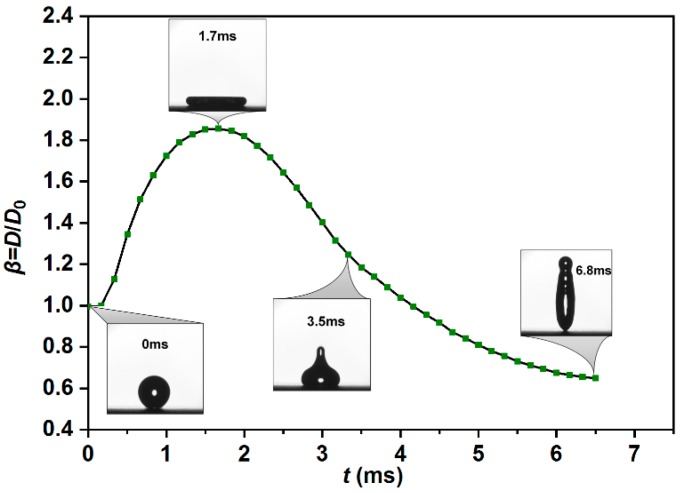
Time evolution of the spreading factor during the first impact on the as-prepared super-hydrophobic surface.

According to previous literature [49], the droplet impacting process could be divided into four phases: kinematic, spreading, relaxation, and wetting/equilibrium phases. Due to the viscosity and surface tension effect, the liquid droplet will bounce off the solid super-hydrophobic surfaces accompanying with the repetition of first three phases. Several bouncing scenarios will be observed before the droplet reaches the equilibrium state. It is known that each spreading or retracting process will lead to energy dissipation because of the viscous friction between the liquid film and the solid surface. Meanwhile, maximum spreading diameter is a key factor to investigate the viscous energy dissipation. Figure 7 shows the temporal evolution of the spreading factor (*β = D/D*_0_) as a function of the spreading time *t* during the first rebounding process (impacting velocity: *V*_0_ = 0.84 m/s; *Re* =2150; *We* = 25). The whole bouncing process can be found in the Supplementary Materials Video S1.

**Figure 8:**
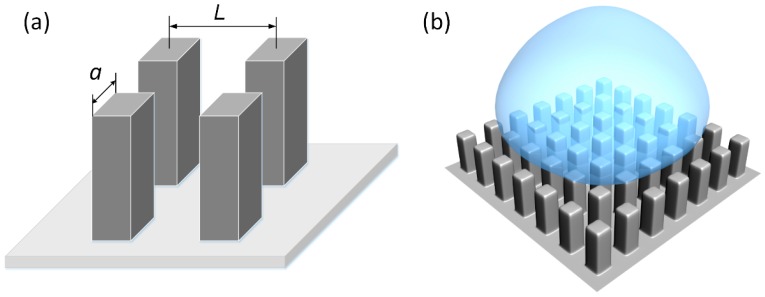
(**a**) The definition length for the micro-pillars and (**b**) the composite wetting state of a droplet during the spreading process.

We assume that the laser-induced super-hydrophobic surface presents inerratic micro-pillar arrays. As shown in Figure 8a, the side length of each pillar is *a*. The center-to-center spacing between two adjacent pillars is *L*. Before the droplet impacting on this solid super-hydrophobic surface, the total energy of the water droplet includes kinetic energy (*E*_k0_) and surface energy (*E*_s0_), which can be given as the following equations:

(1) $$E_{k0}=\frac{1}{12}\pi \rho D_{0}^{3}V_{0}^{2}$$

(2) $$E_{s0}=\pi D_{0}^{2}\sigma$$

where *σ*, *D*_0,_ and *V*_0_ are the droplet surface tension factor, initial droplet diameter, and impacting velocity, respectively.

During the impacting process, the droplet tends to form a liquid film onto the solid super-hydrophobic surface. As previously mentioned, it is assumed that the shape of the liquid film is a cylindrical disk at the maximum spreading state. Meanwhile, the super-hydrophobic surface presents a composite wetting state because part of the liquid film will infiltrate into the micro-pillar arrays (as shown in Figure 8b). Between the solid super-hydrophobic surface and the liquid film, the main contact area is on the top of the micro-pillars (the contact area fraction is *f*_s_). It is easy to obtain that the contact area between the liquid and gas is approximately 1−*f*_s_. At the maximum spreading stage, the reasonable assumption is that the kinetic energy (*E*_k1_) equals zero. Corresponding surface energy of the water droplet can be given as:

(3) $$E_{s1}=\frac{\pi }{4}D_{\max}^{2}\sigma_{\mathrm{LG}}\left(1-\cos\theta_{c}\right)+\frac{2}{3}\pi \left(\frac{D_{0}^{3}}{D_{\max}}\right)\sigma_{\mathrm{LG}}$$

where *σ*_LG_ is the surface tension between liquid and gas interface and *θ*_c_ is APCA of the fabricated super-hydrophobic surface.

During the spreading process, there are two main sources of the viscous dissipation: one comes from the surface interaction between the liquid film and the pillar’s top (*W*_1_), the other comes from the liquid flow among the pillar arrays where the friction is produced between the pillar wall surface and the liquid droplet (*W*_2_). According to Pasandideh-Fard [40], the spreading time for maximum diameter is approximately *t* = 8*D*_0_/(3*V*_0_), and the viscous energy dissipation on a flat solid surface can be described as:

(4) $$W_{1}=\int_{0}^{t}\int_{\Omega }\psi d\Omega dt\approx \psi \Omega t$$

where *ψ* is the function of viscous dissipation (*ψ* = *μ*(*V*_0_/*δ*)^2^), in which *δ* is the boundary layer thickness). Ω is the volume of the droplet (Ω = π*D*_0_^3^/6 ≈ π*D*_max_^2^*h*/4, in which *h* is the height of the cylindrical disk at maximum spreading state). The flow of the drop is approximated by a stagnation flow when impacting on a super-hydrophobic surface. Based on recent literature [50], the boundary layer thickness (*δ* = 2(*μD*_0_/*ρV*_0_)^1/2^) can be suggested to substitute the cylindrical disk height (*h*) in the calculation of *ψ*. Therefore, the dissipation function can be expressed by:

(5) $$\psi =\frac{\rho V_{0}^{3}}{4D_{0}}$$

Thus, considering the area fraction of pillar’s top (*f*_s_ = *a*^2^/*L*^2^), the viscous energy dissipation is assumed to be:

(6) $$W_{1}=\frac{\pi \mu V_{0}D_{\max}^{2}\sqrt{Re}}{3}\frac{a^{2}}{L^{2}}$$

Additionally, the viscous energy dissipation among the liquid flowing through the pillars is due to the friction between the liquid and the pillar wall. We assume that during the spreading, the rid position of the droplet liquid film is *z*. According to the literature [51], the viscous force among the arrays of pillars is

(7) $$F_{\mu }=\alpha \frac{\mu Vbz}{L^{2}\ln\left(L/a\right)}$$

where *α* is the relaxation factor. *V* is the velocity of liquid flow among the array of pillars. *b* is the height of the pillars that were wetted by the liquid film, giving: *b* = *ρV*_0_^2^*L*^2^/(2σ) [52,53]. Using average velocity *V* = *D*_max_*V*_0_/*D*_0_, the viscous force *F_μ_* can be rewritten as:

(8) $$F_{\mu }=\frac{\alpha \mu \rho D_{\max}V_{0}^{3}z}{2\sigma D_{0}\ln\left(L/a\right)}$$

Hence giving:

(9) $$W_{2}=\int_{0}^{R}\frac{\alpha \mu \rho D_{\max}V_{0}^{3}z}{2\sigma D_{0}\ln\left(L/a\right)}zdz=\frac{\alpha \mu \rho D_{\max}^{4}V_{0}^{3}}{48\sigma D_{0}\ln\left(L/a\right)}$$

The following equation can be obtained according to the energy conservation between the pre-impacting state and the maximum spreading state:

(10) $$E_{k0}+E_{s0}=E_{k1}+E_{s1}+W_{1}+W_{2}$$

The maximum spreading factor will be acquired by combining Equations (1)–(3), (6) and (9), which can be described as:

(11) $$\alpha \frac{CaWe}{\ln\left(L/a\right)}\beta_{\max}^{4}+[4\frac{We}{\sqrt{Re}}\frac{a^{2}}{L^{2}}+3\left(1-\cos\theta_{c}\right)]\beta_{\max}^{2}+\frac{8}{\beta_{\max}}-We-12=0$$

The value of relaxation factor *α* was numerically computed at 2.9 in our experiment.

**Figure 9:**
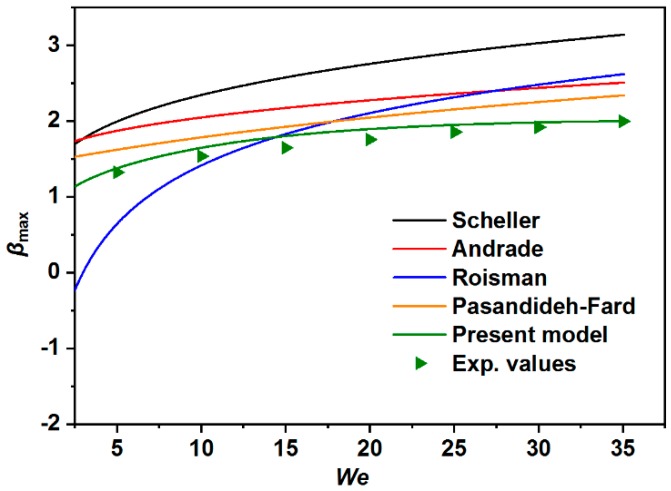
The comparison of experimental data and theoretical predictions of maximum spreading factor. The experiments were performed at various *We* including 5, 10, 15, 20, 25, 30, and 35.

**Figure 10:**
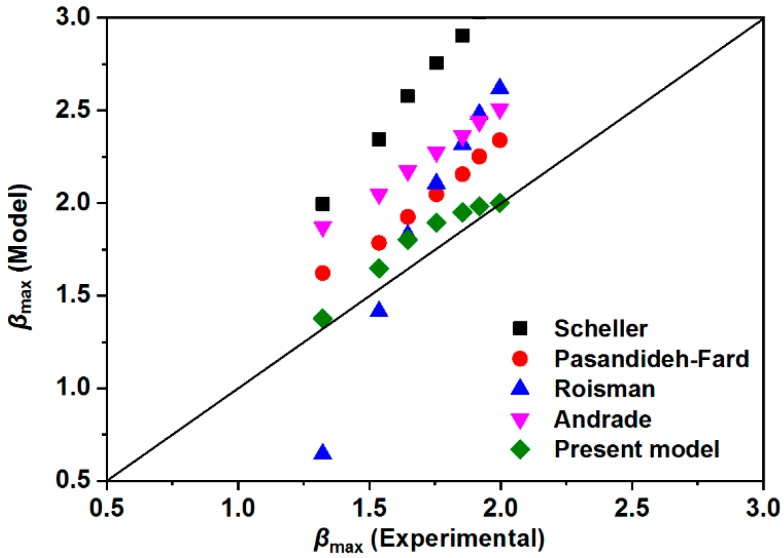
Comparison of the previous models against experimental data.

Figure 9 plots the predictions of theoretical maximum spreading factors obtained from several previous models as well as this present model. The results clearly indicate that none of the existing models could fit our experimental data, while the proposed model in this paper was closely consistent with the obtained experimental data. It is noted from Table 1 that the Andrade model and Roisman model only considered the *Re* and *We* [54,55], and the Scheller formula only took the *Re* and *Oh* into consideration [37]. Although the Pasandideh-Fard et al. [40] introduced one more parameter of advancing the contact angle into their model, the derived formula cannot agree well with the experimental data. Furthermore, Figure 10 compares the previous models against the experimental data for the water droplets. The results approaching the diagonal line demonstrated that the proposed models were in good agreement with the experimental results. In conjunction with Figure 10, the corresponding mean-errors and standard deviations are summarized in Table 2.

It can be concluded that the largest error was presented in the Scheller model, which is an empirical equation based on a flat solid substrate, instead of the super-hydrophobic surface with pillar arrays structure, yielding a mean error of 47.76% with a standard deviation of 0.89. The Andrade model was obtained by optimizing the values of statistical factors from various models including the Scheller model. Although the Andrade model received good accuracy according to their experimental data, this model failed to fit ours, with a mean error of 24.35% and standard deviation of 0.43. Besides, Roisman et al. [55] pointed out that the droplet shape was not a cylindrical disk at the maximum spreading state, and the rim section of the liquid film cannot be ignored. However, most previous models were presented using the supposition of cylindrical disk based on the energy balance concept, such as the Passandideh-Fard model. The results indicate that the Passandideh-Fard model has a better accuracy for our experimental data, with a smaller mean error as well as standard deviation compared to the Roisman model.

**Figure 11:**
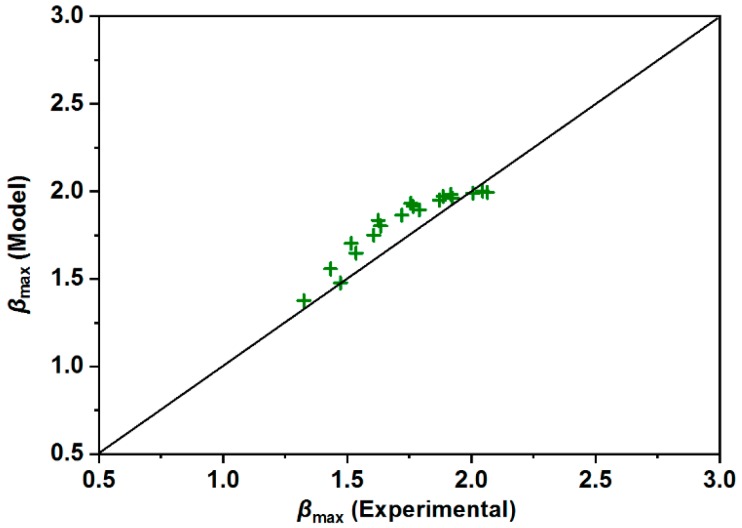
Comparison of additional experimental data against the present model.

It is noted that all of the abovementioned models were obtained by investigating droplet impact on flat solid surfaces, and all the mean errors were above 10% of the experimental data. These models may be limited for super-hydrophobic surfaces possessing unique structures. It is therefore urgent to propose a new model to theoretically simulate the maximum spreading factor on the super-hydrophobic surfaces. Inspired by the Passandideh-Fard model, more parameters, including droplet property (*We*, *Re,* and *Ca*) and surface property (contact angle and geometrical parameters), were taken into consideration. In addition, in terms of the energy conversion between pre-impact state and the maximum spreading state, the viscous energy dissipation was extensively discussed in this current study. The results reveal that the current model was remarkably accurate with a relatively low mean error (4.99%) and standard deviation (0.10). In order to further verify the accuracy of this proposed model, more experiments were carried out based on various impacting velocities. As expected, the good accuracy of the proposed model can be seen in Figure 11 because the small difference between theoretical value and experimental data was observed.

## 4. Conclusions

In summary, a super-hydrophobic aluminum alloy surface with pillar-array structure was successfully obtained by the hybrid laser ablation and post chemical modification. The received super-hydrophobic surface showed a high APCA of 158.2 ± 2.0° and low SA of 3 ± 1°. The surface morphologies and chemical compositions were analyzed to explain the formation of super-hydrophobicity. Moreover, the water droplet impacting process on this super-hydrophobic surface was recorded by a high-speed video camera, and the maximum spreading factor of the water droplet was derived based on the energy conservation concept. Particularly, the viscous energy dissipation caused by liquid flowing among the walls of pillar arrays was considered as well in this current study. The results reveal that the present theoretical predictions were in good accordance with our experimental data when the viscous energy dissipation and geometrical parameters of the pillars were extensively considered.

## Figures and Tables

**Table 1:** Different proposed models for the maximum spreading factor.

Models	Equation
Scheller [37]	$\beta_{\max}=0.61{\left(Re^{2}Oh\right)}^{0.166}$
Andrade [54]	$\beta_{\max}=1.28+0.071We^{1/4}Re^{1/4}$
Roisman [55]	$\beta_{\max}=0.87Re^{1/5}-0.40Re^{2/5}We^{-1/2}$
Pasandideh-Fard [40] ^1^	$\beta_{\max}=\sqrt{\frac{We+12}{3(1-\cos\theta_{a})+4(We+\sqrt{Re})}}$
Present model	$\alpha \frac{CaWe}{\ln(L/a)}\beta_{\max}^{4}+[4\frac{We}{\sqrt{Re}}\frac{a^{2}}{L^{2}}+3(1-\cos\theta_{c})]\beta_{\max}^{2}+\frac{8}{\beta_{\max}}-We-12=0$

^1^*θ_a_* represents advancing contact angle.

**Table 2:** Summary of mean-errors and standard deviations of different prediction model.

Model	Mean Error (%)	Standard Deviation
Scheller	47.76	0.89
Andrade	24.35	0.43
Roisman	22.09	0.44
Pasandideh-Fard	11.72	0.22
Present model	4.99	0.10

## Supplementary Materials

The following are available online at https://www.mdpi.com/1996-1944/12/5/765/s1, Video S1: High-speed visualization of the droplet impact on the as-prepared super-hydrophobic surface at *We* = 25.
